# Supplementary material for: Bacterial Populations in International Artisanal Kefirs
Source: Microorganisms. 2020 Aug 29;8(9):1318. doi: 10.3390/microorganisms8091318 (PMC7565184; doi:10.3390/microorganisms8091318)
Supplement: Supplementary file 1 [file microorganisms-08-01318-s001.pdf]

Supplementary Materials

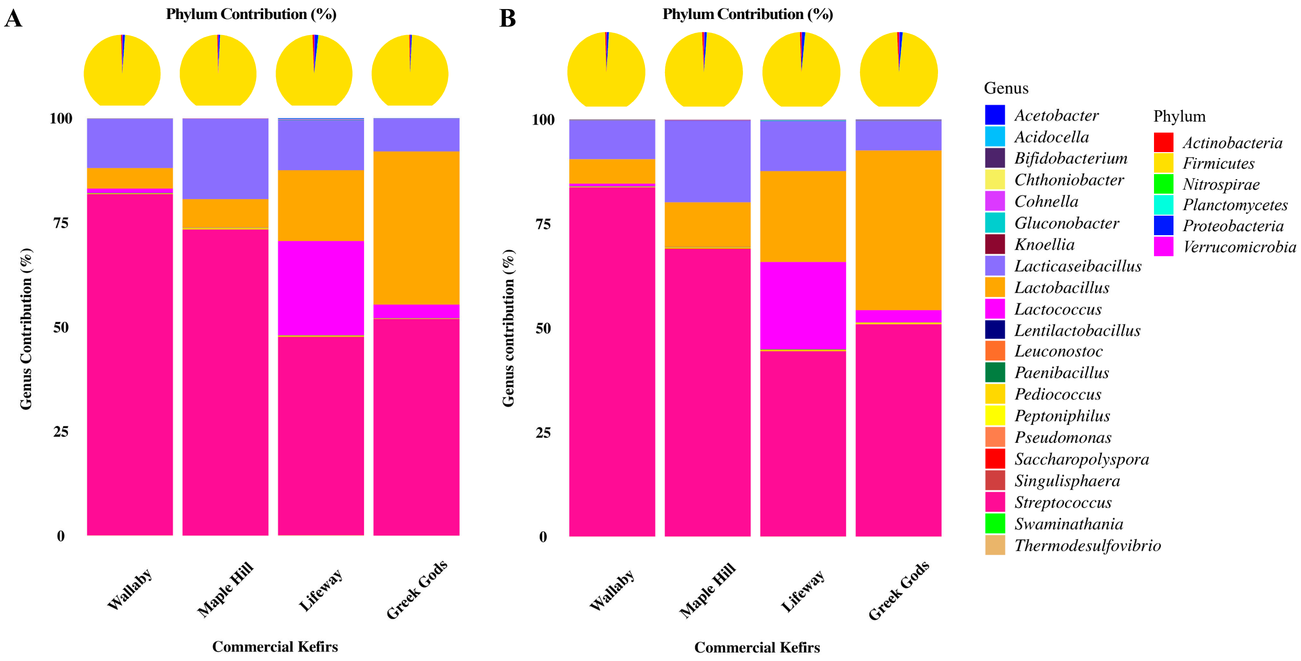

Figure S1. Relative abundances of bacterial phylum (Pie charts) and an aggregation of the 21 most abundant bacterial genera (column charts) for the V1–V3 (**A**) and V3–V4 (**B**) regions of 16S rRNA genes. Commercial kefir controls: Lifeway (plain), The Greek Gods (plain), Wallaby (organic plain), and Maple Hill (organic plain).
